# Deep learning data augmentation for Raman spectroscopy cancer tissue classification

**DOI:** 10.1038/s41598-021-02687-0

**Published:** 2021-12-13

**Authors:** Man Wu, Shuwen Wang, Shirui Pan, Andrew C. Terentis, John Strasswimmer, Xingquan Zhu

**Affiliations:** 1 Department of Electrical Engineering and Computer Science, Florida Atlantic University, Boca Raton, USA; 2 Faculty of Information Technology, Monash University, Melbourne, Australia; 3 Department of Chemistry and Biochemistry, Florida Atlantic University, Boca Raton, USA; 4 Department of Integrated Medical Science, Florida Atlantic University, Boca Raton, USA

**Keywords:** Basal cell carcinoma, Melanoma, Squamous cell carcinoma

## Abstract

Recently, Raman Spectroscopy (RS) was demonstrated to be a non-destructive way of cancer diagnosis, due to the uniqueness of RS measurements in revealing molecular biochemical changes between cancerous vs. normal tissues and cells. In order to design computational approaches for cancer detection, the quality and quantity of tissue samples for RS are important for accurate prediction. In reality, however, obtaining skin cancer samples is difficult and expensive due to privacy and other constraints. With a small number of samples, the training of the classifier is difficult, and often results in overfitting. Therefore, it is important to have more samples to better train classifiers for accurate cancer tissue classification. To overcome these limitations, this paper presents a novel generative adversarial network based skin cancer tissue classification framework. Specifically, we design a data augmentation module that employs a Generative Adversarial Network (GAN) to generate synthetic RS data resembling the training data classes. The original tissue samples and the generated data are concatenated to train classification modules. Experiments on real-world RS data demonstrate that (1) data augmentation can help improve skin cancer tissue classification accuracy, and (2) generative adversarial network can be used to generate reliable synthetic Raman spectroscopic data.

## Introduction

Skin cancer, one of the most common cancers across the world, accounts for more than 40% of global total cancer cases, in which the top three skin cancer types are basal cell skin cancer (BCC), squamous cell skin cancer (SCC), and non-melanoma skin cancer (NMSC)^1,2^. According to the American Academy of Dermatology Association (AAD), the daily number of diagnosed skin cancer cases in the US is approximately 9500 and one in five Americans is estimated to develop skin cancer in their lifetime^3^. Although surgical removal is the optimal method for skin cancer diagnose and treatment, current in situ methods can hardly differentiate cancer from normal skin^2^. In addition, the surgical process is time-consuming and patients may suffer from heavy financial burden^4^. In contrast, the vibrational modes of molecules can be easily and correctly analyzed with Raman Spectroscopy (RS) technique, which is able to detect differences in the molecular structures of proteins, lipids and pigments of both tumor and normal tissues^5,6^.

Raman Spectroscopy (RS) is a non-destructive in situ spectroscopic chemical analysis technique that provides detailed information about chemical structure, phase and polymorphism, crystallinity and molecular interaction. RS is an inelastic light scattering technique producing scattered photons either lower in energy (Stokes) or higher in energy (anti-Stokes) than the exciting photons. The energy shifts of the photons correspond to the energies of vibrations of molecules in the sample, thus providing detailed chemical structure information revealing chemical compositions of cells and tissues^7^.

**Figure 1:**
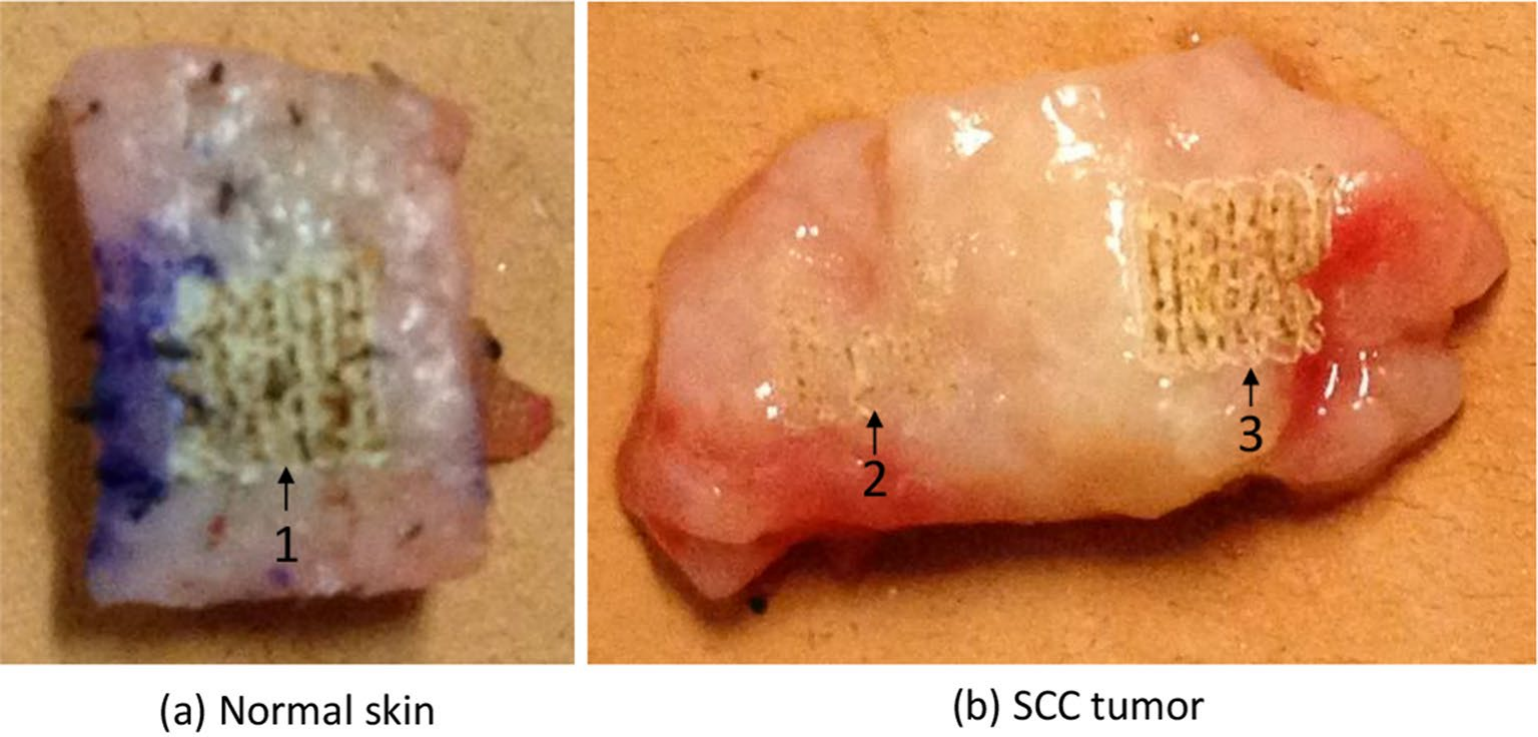
Examples of normal (**a**) vs. squamous cell carcinoma tissue (**b**) specimens. Square regions indicated by the arrows were treated with a high-powered IR laser to ablate the tissue surface. Raman spectra were collected from both ablated and non-ablated regions of the samples. The numbers, 1, 2, and 3, indicate each distinct ablation treatment area.

Using RS and machine learning for skin cancer detection has been studied previously^8^, where spectral classification is used to classify BCC based on tissue samples from 55 patient, among which logistic regression classifier using five canonical spectral features obtained from rank-reduced multiclass linear discriminant analysis outperforms the rest classifiers. Previously, we also employed Principal Component Analysis (PCA) to differentiate non-melanoma skin cancer (NMSC) from normal skin with the combination of RS and high-powered ${\hbox {CO}}_2$ laser to ablate the tissue surface (an example of laser treatment is shown in Fig. 1). In our study^9^, the Raman spectra are collected from both ${\hbox {CO}}_2$ treated and untreated samples, and are further used to train a binary logistic regression model to distinguish normal from diseased tissues. The comparative study validates the effectiveness of Raman Spectroscopy-high powered ${\hbox {CO}}_2$ method in clinical skin cancer treatment.

Despite of the promising properties, RS suffers from weak signals due to inherent noise. Shot noise and fluorescence’s baseline are the most commonly found noise in RS signals. The shot noise is the results of the unavoidable statistical nature of light while the fluorescence’s baseline can mask Raman bands with a higher amplitude. Although several denoising method have been proposed, noise can hardly be effectively removed from the signal to limit the negative impact of the noise, which will damage the integrity of the Raman spectrum^10,11^. Discrete wavelet transform (DWT) has been applied to separate shot noise, fluorescence’s baseline and informative Raman peaks by decomposing the spectrum into a set of wavelet coefficient and scale coefficient^12,13^. Other advanced methods adapted based on DWT such as adaptive lifting wavelet transform (ALWT) are also proved effective in removing noise from Raman spectrum^14^. In our previous study^9^, samples were processed with laser ablation, as shown in the top row in Fig. 1. The study systematically varied the ablation level and examined its impact, which showed that Raman spectral features from normal and cancerous tissue did not significantly correlate with ablation treatment level (therefore, in this study, we combine both laser treated and untreated samples to maximize number of available samples).

Another challenge in RS based skin cancer detection is that the data are often ill-posed, because Raman spectrum has a background resulted from skin fluorescence, both the spectra and correlation can be introduced with variance. Apart from that, the number of frequency components in Raman spectrum is normally $10^3$–$10^4$, indicating a large dimension of features. In addition, for cancer diagnose, few valuable samples are available due to privacy and other constraints^15,16^. The data scarcity, combined with high data dimensionality, present difficulties for deep learning algorithms.

**Figure 2:**
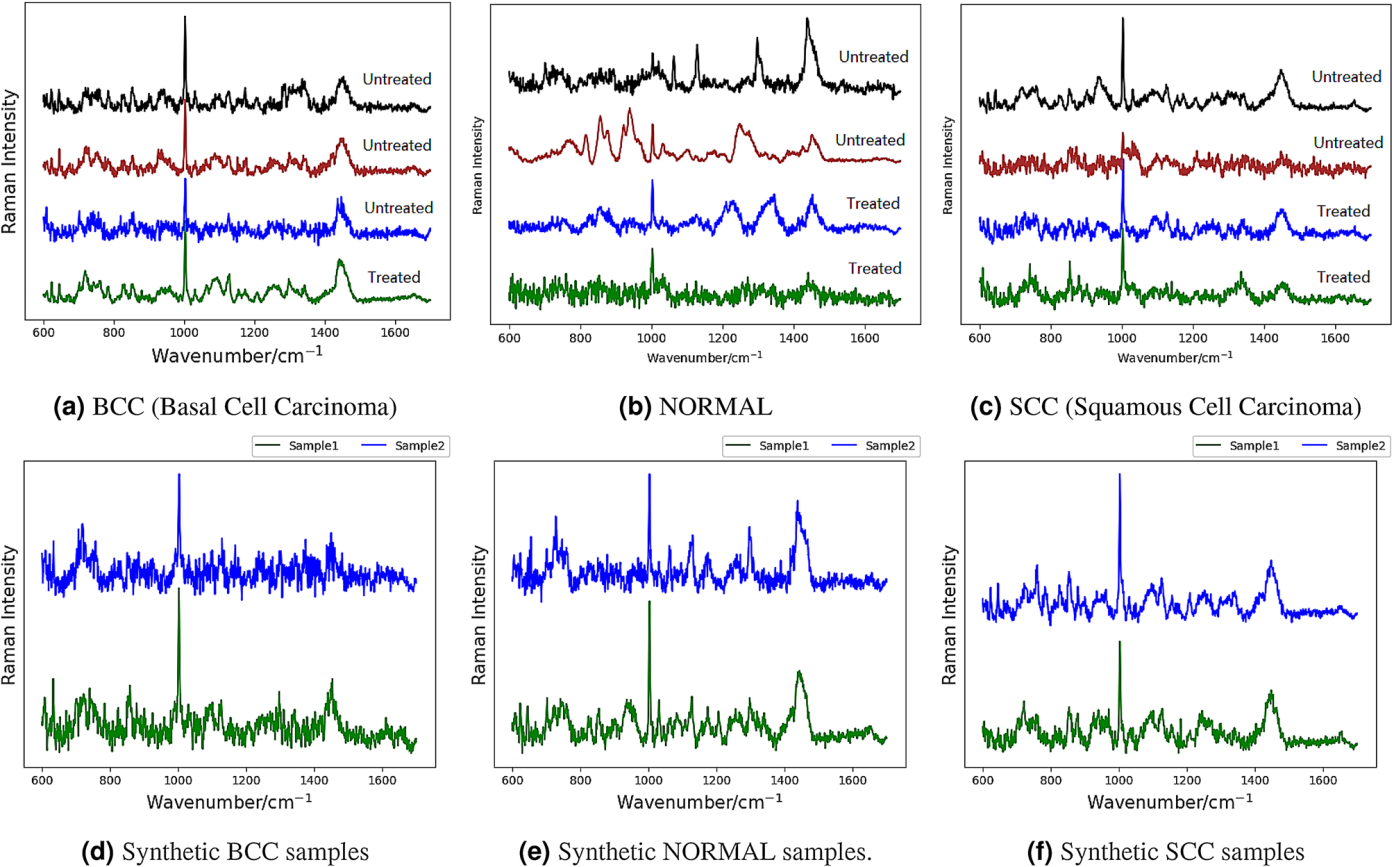
Top row (**a**–**c**) Genuine Raman spectra measured from a dataset with three categories: BCC (basal cell carcinoma), NORMAL, and SCC (squamous cell carcinoma), which are representative of the range of spectra measured in this work. Each sample has 1608 dimensions, where each dimension correspond to the wavelength number of the Raman shift (ranging from 600.237 to 1699.39 ${\hbox {cm}}^{-1}$). The value of each dimension represents the Raman intensity. “Treated” means that the sample has been treated using a high-powered IR laser to ablate the tissue surface, or no laser treatment otherwise (“Untreated”). Each colored curve represents one RS sample. Bottom Row (**d**–**f**) Synthetic Raman spectra samples generated using GAN for each cancer category (BCC, NORMAL, and SCC). Each category has two generated RS samples.

The above challenges motivate our research to use Generative Adversarial Network (GAN) based data augmentation method to increase the sample size for RS based skin cancer detection. GAN is a method to generate data resemble to the training data, using two deep networks, the generator, and the discriminator. Candidates are generated by generative network while the discriminative network evaluates. Through the iterative generation and evaluation process, new data, with the same statistics as the original data set, are generated. Examples of GAN generated RS samples are shown in the bottom row in Fig. 2.

Using data augmentation, new samples for each category of the original data are generated in order to improve the performance of the downstream classifiers. For data classification part, a deep convolutional neural network (CNN) is designed as the core module to compare with other baseline models. We employ CNN as the classifier for RS cancer tissue classification, mainly because that CNN has unique convolutional filters to explore correlation of the signal and learn patterns to differentiate signals between different classes.

### Contribution

In this paper, we address the data scarcity of RS cancer tissue classification by using deep learning based data augmentation and classification. This study has three main contributions as follows:

- **Deep learning to tackle RS sample scarcity** In cancer diagnosis, due to privacy and other restrictions, very few valuable samples are available for training reliable models. Our research proposes solutions to tackle this challenge by applying generative adversarial network to generate synthetic RS samples. We validate and compare the effectiveness of this approach vs. other baselines.
- **RS sample augmentation approaches** We design two sample augmentation approaches, balanced data augmentation and stratified data augmentation, to evaluate how augmented samples should be integrated for learning accurate models.
- **Deep learning for RS cancer tissue classification** By leveraging sample augmentation, we propose a deep learning based framework and compare its performance using a variety of deep learning models, including CNN and Long Short Term Memory Networks (LSTM), for Raman spectroscopy cancer tissue classification.

## Related work

### Raman spectroscopy for skin cancer diagnosis

Raman spectroscopy has been considered as a promising non-destructive optical technique characterizing the tissue at the molecular level^17^. Recent studies reported that Raman spectroscopy (RS) is beneficial to diagnose and study the evolution of human malignancies both in vitro and in vivo^18^. It has been widely proved that Raman spectroscopy can be applied to distinguish skin cancers from normal skin tissues for accurate medical diagnosis^19^.

Several researches have utilized RS to diagnose skin cancer. Harvey et al.^20^ evaluated the application of an integrated real-time system of RS for in vivo skin cancer diagnosis. The performance, in terms of ROC (Receiver Operating Characteristic) curve, can be dramatically increased to 0.879 by using a primary module with generic discriminant analysis in lesion classification. Lieber et al.^17^ measured Raman spectrum of 21 suspected non-melanoma skin cancers and detected lesions from normal skin yielding 91$\%$ specificity and 100$\%$ sensitivity. A 95$\%$ separation accuracy between normal skin and BCC is achieved by Choi et al.^21^ with Confocal Raman spectra obtained from various skin depths. The reserach of Fox et al.^9^ shows that RS classification accuracy is not negatively affected by the ablation process and also beneficial to tumor border demarcation. A hybrid fluorescence Raman approach or a non-linear Raman technique can be used to reduce imaging time.

Due to weak Raman intensity caused by the poor scattering efficiency, PCA analysis or Neural Network is preferred to distinguish skin cancer tissue from normal tissue^21^. Therefore, deep learning methods have been integrated in our study.

### Deep learning for biosignal processing

Analysis and interpretation of biological signals are highly intricate research tasks. Deep learning extracts signal’s features automatically from raw data with a better performance when amounts of data are available for learning, while traditional machine learning methods to understand and translate biological signals are based on hand-engineered features. Biosignals and deep learning are often utilized to solve specific application, such as health status monitoring, emotion detection, analysis and classification of human gestures, diagnosis for illness and so on. Among all applications, we focus on the support for diagnosis using deep learning.

An image of a skin lesion was successfully transformed into the probability distribution of clinical dermatoses by Andre et al.^22^ with CNN. It achieved similar performance with all tested experts’ judgment on the identification of common cancers and the identification of the deadliest skin cancer. Budak et al.^23^ proposed an end-to-end system based on fully convolutional network (FCN) and bidirectional long short term memory (BiLSTM) for detections of breast cancer. A five-fold cross-validation technique was considered to calculate accuracy metric, which showed that their proposed method was better than those preliminary reported results. Mahbod et al.^24^ used three pretrained deep models, namely AlexNet, VGG16 and ResNet-18, as deep feature generators. The extracted features were sent to support vector machine classifiers, yielding an area under ROC curve of 83.83$\%$ for melanoma classification and of 97.55$\%$ for seborrheic keratosis classification. Transfer learning method is usually applied in the deep learning structures as well.

All the detecting systems by using deep learning mentioned above are based on large amounts of data. In terms of augmenting image sets, GAN is widely applied to overcome the sensitivity of synthetic data samples for the cancer data classification^25^. A skin lesion style-based generative adversarial networks (GANs) model is proposed in^26^ and proved to be effective for generating skin lesion images with high resolution and abundant diversity. Compared to the prior CNN model, classification indexes like accuracy, sensitivity, specificity, average precision and balanced multi-class accuracy have been improved by 1.6$\%$, 24.4$\%$, 3.6$\%$, 23.2$\%$ and 5.6$\%$ respectively. A method for synthesizing insect pest training images through GAN is put forward to enhance CNN’s performances in the Ref.^27^. The F1 density of the classifier model trained with GAN-based augmentation is 0.95, outperforming the models trained with traditionally augmented images with an F1-score of 0.92. There is sufficient evidence to prove that by using GAN-based enhancement methods, deep learning classification models have better performances than using traditional enhancement methods.

## Problem definition and overall framework

### Problem statement

In this paper, skin cancer tissue classification task is defined as a multi-class classification problem. In this work, we use the tissue dataset from a previous study^9^, which consists of three tissue categories: BCC (basal cell carcinoma), SCC (squamous cell carcinoma) and NORMAL. Using RS process, each sample is represented by 1608 dimensions, denoting the frequency of the Raman shift (ranging from 600.237 to 1699.39 wavenumber ${\hbox {cm}}^{-1}$), and the value of each dimension represents the Raman intensity. Examples of RS spectra (intensity vs. ${\hbox {cm}}^{-1}$) are reported in the top row in Fig. 2.

Let $X =\left\{ {\mathbf {x}}^{1}, \ldots , {\mathbf {x}}^{n}\right\}$ denotes the given RS dataset, where *n* is the number of samples in the dataset, ${\mathbf {x}}^{i} \in \mathbb {R}^{1\times d}$, *d* is the dimension of each sample. Because we use RS intensity at each wavelength number (${\hbox {cm}}^{-1}$) position as feature values to present each sample, the total feature dimension is $d=1608$.

Each sample ${\mathbf {x}}^{i}$ is associated with a ground-truth label ${\fancyscript {Y}} \in \{{\text {BCC}}, {\text {Normal}}, {\text {SCC}}\}$. The goal of skin cancer tissue classification is to learn a projection function: ${\fancyscript {F}}({\mathbf {x}}^{i}) \rightarrow {\fancyscript {Y}}$

### Overall framework

**Figure 3:**
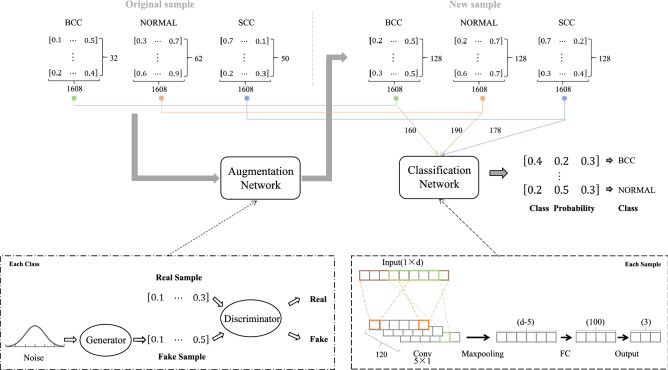
Illustration of the generative adversarial network based data augmentation for Raman Spectroscopy cancer tissue classification framework.

Our framework introduces a novel generative adversarial network based medical data augmentation for Raman Spectroscopy cancer tissue classification, as shown in Fig. 3, mainly consists of following two components:

- **Data augmentation module** In order to increase the number of training samples, we employ a Generative Adversarial Network to generate synthetic samples for each class, BCC, SCC, and Normal, respectively. This process will generate different types of samples for data augmentation.
- **Data classification module** Combining original samples and GAN generated sample, the data classifier will learn discriminative models to determine the category of each sample. In our study, we exploit using a deep convolutional neural network (CNN) to classify each sample into respective category, and also comparatively study other rival methods, such as logistic regression (LR), support vector machines (SVM).

## Methodology

Figure 3 shows the proposed framework for RS based cancer tissue classification, and the detailed algorithmic procedures are reported in Algorithms 1 and 2. Overall, the framework includes two main modules: Data augmentation module and data classification module. The former will learn to generate synthetic samples for each class, and the latter will learn to classify test samples into correct categories.

### Data augmentation module

In order to solve the small sample size problem, we propose to use Generative Adversarial Network (GAN) to investigate data augmentation. Generative Adversarial Network(GAN) has been implemented to synthesize high quality data for adding training data in several studies^28^. As shown in the lower left dashed rectangular box in Fig. 3, the GAN includes two major building blocks: generator and discriminator. The two blocks are both consists of multilayer perceptrons.

- The generator *G* is to generate fake samples from the latent vector *z*. The generator can be thought of as analogous to a team counterfeiters, trying to produce fake samples and try to induce the discriminator to give the generated sample a higher score.
- The discriminator *D* is analogous to the police, which tries to discriminate between the original data and the generated samples.

The generator and discriminator are running in an adversarial way to improve each other. Specifically, the discriminator *D* tries to learn the original data and guides the generator by sending feedback about the generated synthetic samples. The generator *G* learns from the feedback and tries to generate new samples which are very close to the original data. The discriminator *D* is trained to maximize the probability of distinguishing original samples from samples generated from the generator *G* (i.e. correctly predict whether a sample is generated or not) The loss function of the discriminator *D* can be expressed as:

1 $$\begin{aligned} \max _{D}V(G,D)=E_{x\sim p_{d}}[\log D(x)]+E_{x\sim p_{z}}[\log (1-D(G(z)))] \end{aligned}$$

Simultaneously, the generator *G* is trained to minimize $\log (1-D(G(z)))$, which tries to make generate samples resemble to the genuine training samples, as much as possible. The loss function of the generator *G* can be expressed as:

2 $$\begin{aligned} \min _{G}V(G,D)=E_{x\sim p_{z}}[\log (1-D(G(z)))] \end{aligned}$$

In other words, *D* and *G* are trained by a two-player minimax game with the loss function:

3 $$\begin{aligned} \min _{G}\max _{D}V(G,D)=E_{x\sim p_{d}}[\log D(x)]+E_{x\sim p_{z}}[\log (1-D(G(z)))] \end{aligned}$$

In Fig. 2 (bottom row), we visualize synthetic RS samples generated using GAN, with respect to each tissue category. The examples show that samples generated from GAN are very similar to the genuine RS data (the top row), which demonstrates the potential effectiveness of the data augmentation. In the experiments, we will also show that synthetic data are not only visually similar, but also preserve similar feature representations/distributions, as genuine examples.

### Data classification module

From the data augmentation module, we can obtain a set of new samples for each category of the original data, which will be used to enhance the performance of the classifier. In our classification module, we employ a deep convolutional neural network (CNN) as the core module.

Let $x^{i} \in \mathbb {R}^d$ be the *d*-dimensional feature corresponding to the *i*th sample. For each sample $x^{i}$, we apply a 1-D convolution with a width-*k* kernel to produce a new feature.

4 $$\begin{aligned} f^{i}_{l}={\text {ReLU}}({\mathbf {w}}*x^{i}_{l:l+k-1}+b) \end{aligned}$$

where $l \in \{1, 2, \ldots , d\}$, *w* is a filter, and *b* is a bias term. This filter is applied to each possible window of features in the *i*-th sample to produce a feature map $\mathbf {{f}^{i}}\in \mathbb {R}^{d-k+1}$, where $\mathbf {{f}^{i}}=[f^{i}_{1},f^{i}_{2}, \ldots , f^{i}_{d-k+1}]$.

**Figure 4:**
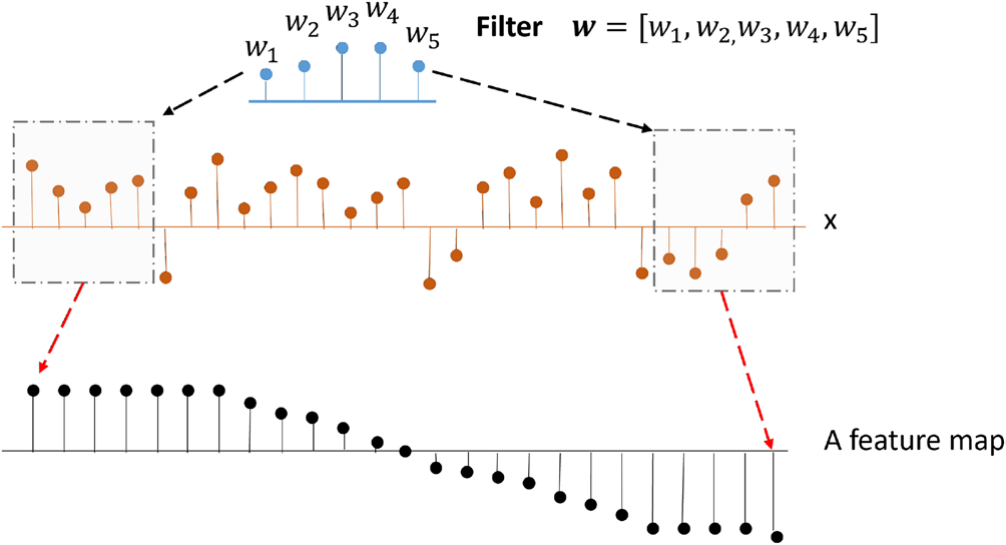
1-D convolution process. The convolution kernel (filter) performs a convolution operation on each location of the input signal ${\mathbf {x}}$, where a window with size *k* (the dashed rectangular box) is used to extract local signal for analysis. The result of each convolution calculation outputs a point as shown in the feature map (the lower panel). The convolution kernel will slide, from left to right, through the signal ${\mathbf {x}}$ to generate feature map.

In order to obtain multiple features, our classification model uses *r* filters. So we can obtain a feature matrix $\mathbf {{F}^{i}}\in \mathbb {R}^{r\times (d-k+1)}$ for the *i*-th sample, with $\mathbf {{F}^{i}}=[\mathbf {{f}^{i}_{1}},\mathbf {{f}^{i}_{2}}, \ldots , \mathbf {{f}^{i}_{r}}]$. In this paper, a filter is a one dimensional weight vector $\mathbf{w }=[w_1,w_2,\ldots ,w_k]$, which will be learned during the training process. An example of convolution process is shown in Fig. 4, where a filter (with $k=5$) is applied to each possible window of the input signal to produce a feature map $\mathbf {{f}^{i}}$. It is worth noting that weight values of each filter are unknown, and are learned during the model training phase.

After the convolution process, we apply 1D Max pooling to the feature map matrix $\mathbf {{F}^{i}}$ to obtain the feature corresponding to all filters:

5 $$\begin{aligned} \tilde{\mathbf {{F}^{i}}}=\text {Maxpooling}(\mathbf {{F}^{i}}) \end{aligned}$$

where $\tilde{\mathbf {{F}^{i}}} \in \mathbb {R}^{(d-k+1)}$. The feature $\tilde{\mathbf {{F}^{i}}}$ is passed to a fully connected layer with softmax function to predict the label of *i*-th sample.

6 $$\begin{aligned} \begin{array}{cl} {\hat{y}^i}=\sigma (W^o*\tilde{\mathbf {{F}^{i}}}+b^o) \end{array} \end{aligned}$$

where ${\mathbf {W}}^o$ and $b^o$denote weight values and bias of the output layer, $\sigma (.)$ is the softmax activation function, and $\hat{y}^i$ denotes the predicted probabilities of the *i*th sample.

### Classification loss

The classification loss ${\fancyscript {L}}(\Theta )$ is to minimize the cross-entropy for the labeled data:

7 $$\begin{aligned} {\fancyscript {L}}(\Theta )= -\frac{1}{N}\sum _{i=1}^{N}y^{i}\mathsf {log}(\hat{y}^{i}), \end{aligned}$$

where N is the sum of the number of original samples and generated samples. $y^{i}$ denotes the label of the *i*-th sample, $\hat{y}^{i}$ is the prediction of the classifier generated from Eq. (6).

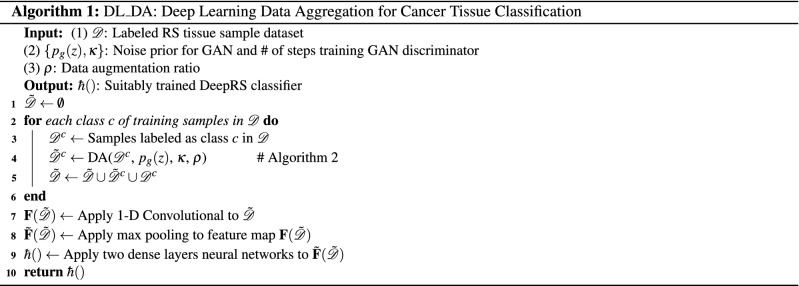

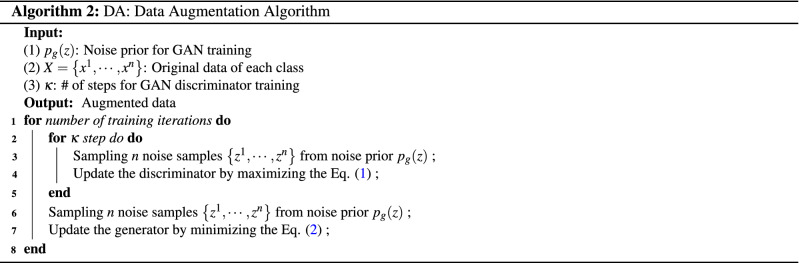





### Data augmentation algorithm description

Algorithm 1 lists the detailed procedures of the proposed algorithm for RS based cancer tissue classification. Algorithm 2 lists the RSDA data augmentation module, which is used in Algorithm 1.

Given a noise prior $p_{g}(z)$ and original data of each class $X = \left\{ x^1, \ldots , x^n\right\}$, the goal of RSDA data augmentation module is to generate new samples for each class of orginal data. The process is alternated between *k* steps of optimizing discriminator *D* and one step of optimizing generator *G*. Firstly, we maximize the loss function of discriminator (Step 3–4). After *k* steps, we then minimize the loss function of generator (Step 6–7). After the training process, we add the generated new samples to the original dataset to expand the training dataset to learn convolutional neural networks for classification.

### Data augmentation approaches

In order to study the impact of data augmentation on the classifier performance, we propose two augmentation approaches as follows:

- **Balanced data augmentation **(${\hbox {DA}}_b$) This approach intends to introduce same amount of synthetic samples to each class. Using data augmentation, we generate $n'$ new samples for each class (each sample has 1608 dimensions). Therefore, the number of BCC, NORMAL and SCC samples increase to $n'+36$, $n'+63$ and $n'+50$, respectively. After that, all samples are combined to form a training dataset with $3*n'+149$ samples.
- **Stratified data augmentation **($\hbox {DA}_s$) This approach intends to maintain the same class prior probability during the data augmentation process, by generating a different number of augmentation samples for each category according to the proportion *m* between different categories of the original data. Accordingly, the number of augmentation samples is $m*36$ for BBC, $m*63$ for NORMAL, and $m*50$ for SCC class, respectively.

## Experiments

### Experimental setup

**Benchmark datasets** Benchmark data used for the experiments were originally collected from Strasswimmer Mohs Surgery, Delray Beach, FL, where the data are used for a study to validate impact of laser treatment for SCC vs. normal tissue classification^9^. Further information about the tissue preparation, treatment, and laser used for the Raman measurements are also detailed in the publication^9^. In our study, all RS data used in the experiments were reprocessed and de-identified. From the processed data, we create a dataset with three categories: BCC (basal cell carcinoma), NORMAL and SCC (squamous cell carcinoma), each contains 36, 63, and 50 RS samples, respectively. Each sample has 1608 dimensions, representing the wavelength numbers of the Raman shift (ranging from 600.237 to 1699.39 ${\hbox {cm}}^{-1}$). The value of each dimension represents the Raman intensity. The details of the benchmark RS data are reported in Table 1.

**Table 1:** Statistics of the benchmark RS data.

Category	# of Treated	# of UNTreated	# of All data
BCC	2	34	36
Normal	28	35	63
SCC	20	30	50

**Data augmentation** In our experiments, for Balanced Data Augmentation, we set four different sample sizes, $n' = \{128, 256, 512, 1,024\}$, respectively, and set five different proportion sizes, $m = \{1, 2, 4, 6, 8\}$, respectively, for Stratified Data Augmentation, to validate the impact of the different augmentation sample sizes on the classification results.

In GAN, the generator consists of one fully-connected layer with 100 hidden units and three fully-connected layers with 64 hidden units, using Rectified Linear unit (ReLU) activation functions. Furthermore, each fully-connected layer is followed by a Batch Normalization. The discriminator consists of one fully-connected layer with 1608 hidden units and two fully-connected layers with 64 hidden units, using LeakyReLU activation functions.

**Evaluation metrics** The skin cancer tissue classification is a multi-class classification task, whose evaluation measure is commonly the Accuracy metric. However, when datasets suffer from class imbalance, it goes less reliable. Therefore, in addition to the Accuracy metric, we add Macro-F1 and Area Under Curve (AUC) metrics. For all experiments, we use leave-one-out cross validation on total samples throughout the experiments.

### Baselines

We implement following baselines for comparisons to demonstrate the effectiveness of our proposed model.

- **LR** is a Logistic Regression model, which directly feed the sample features into the Softmax classifier to determine the category.
- **PCA_LR** first uses Principal Component Analysis (PCA) to reduce the input features to 100 dimensions, and then trains logistic regression classifier for classification.
- **LR**_$\hbox {DA}_x$ applies linear regression (LR) to the augmentation of the original and synthetic data, and train an LR model for classification. We will study both balanced ($x=b$) and stratified augmentation ($x=s$) in the experiments.
- **SVM** is a support vector machine classifier learned in the original feature space.
- **PCA_SVM** uses PCA to reduce input features to 100 dimensions, then trains SVM classifiers for classification.
- **SVM**_${\hbox {DA}}_x$ applies SVM to the augmentation of the original and synthetic data, using both balanced augmentation ($x=b$) and stratified augmentation ($x=s$), and trains an SVM model for classification.
- **MLP** is a Multilayer Perceptron network, which consists of multiple fully connected layers.
- **MLP**_${\hbox {DA}}_x$ applies MLP to the augmentation of the original and synthetic data, using both balanced augmentation ($x=b$) and stratified augmentation ($x=s$), and train an MLP model for classification.
- **LSTM** is a long short-term memory neural network capable of learning features from long sequence inputs.
- **LSTM**_${\hbox {DA}}_x$ applies LSTM to learn feature representations from augmentation of the original and synthetic data, using both balanced augmentation ($x=b$) and stratified augmentation ($x=s$). Finally, we deploy a fully connected layer with corresponding activation function to predict the class of samples.
- **CNN** uses a convolutional neural network to learn sample representations by sliding windows on sample features.
- **CNN_SMOTE** uses a convolutional neural network to learn sample representations from the augmentation of the original and synthetic data. The data augmentation method uses Synthetic Minority Oversampling Technique (SMOTE)^29^. The SMOTE method is based on the *k* nearest neighbor sample points of each sample point, and randomly selects *N* neighboring points to multiply the difference by a threshold in the range of [0,1] to achieve the purpose of data enhancement.
- **DL**_${\hbox {DA}}_x$ is our deep learning model for RS based cancer tissue classification. We use CNN to learn feature representations from the augmentation of the original and synthetic data, using both balanced augmentation ($x=b$) and stratified augmentation ($x=s$), followed by a pooling and a dense layer with the softmax function, to predict the class label of each sample.

### Implementation details

For training, we use 500 epochs for all models. For data augmentation module training, we use the Adam optimizer with the initial learning rate of 0.0002 to train the GAN model, and then generate a certain number of augmentation samples for each class. In GAN, the generator is composed of one fully-connected layer with 100 hidden units and three fully-connected layers with 64 hidden units, which are activated using Rectified Linear unit (ReLU). Furthermore, each fully-connected layer is followed by a Batch Normalization. The discriminator is composed of one fully-connected layer with 1,608 hidden units and two fully-connected layers with 64 hidden units, activated using LeakyReLU. For classification module of CNN, the number of convolution filters *r* and their width/size *k* are set to 120 and 5, respectively. The classification module of LR has one fully-connected layer with the number of hidden units 3, which is activated using Softmax. For classification module of SVM, the *Maxiter* of LinearSVC is set to 500. The MLP classification module has three fully-connected layers activated by Rectified Linear unit (Relu), and the hidden units of each fully connected layer are set to 100, 32, and 3 respectively. The LSTM classification model consists of an LSTM layer with 100 hidden units and three fully-connected layers with 10, 500, and 3 hidden units, respectively. For each network, we use a fixed learning rate $1e^{-3}$. All deep learning algorithms are implemented using Tensorflow and are trained with Adam optimizer.

### Results and analysis

Tables 2 and 3 reports the performance comparisons (Accuracy, Macro F1 score, and AUC) between the proposed method against baselines, using balanced data augmentation (Table 2) and stratified data augmentation (Table 3), respectively. From the results, we have following observations:

1. Most deep network models, such as CNN and LSTM, outperform traditional machine learning models, like LR and SVM. This demonstrates that deep learning methods, even with limited training samples, can learn better hidden representations of samples. This is because that deep learning can better leverage correlation in the RS signals to learn patterns, whereas LR and SVM take raw RS spectra as features, where feature correlation will deteriorate the classifier performance.
2. Models with data augmentation (e.g., MLP_${\hbox {DA}}_b$, CNN_${\hbox {DA}}_b$, LSTM_${\hbox {DA}}_b$) have better performances than single classifier models (e.g., MLP, CNN, LSTM), which shows that the augmentation module can improve the performance of classifier models.
3. In the ability of improving classification accuracy, CNN_${\hbox {DA}}_b$ and LSTM_${\hbox {DA}}_b$ is better than SVM_${\hbox {DA}}_b$ and LR_${\hbox {DA}}_b$, confirming that the addition of augmentation module will be more effective for deep network models. This is probably because the deep network needs more data to satisfy the requirements of the model.
4. Compared with all baselines, our proposed DL_${\hbox {DA}}_b$ achieves the best performance and outperforms other classification method with augmentation module in most cases. The superiority of DL_${\hbox {DA}}_b$ is attributed to its data augmentation which provides new samples to help the classification model consistently perform well when few samples are available for training.
5. Compared with CNN_SMOTE, another data augmentation method, our proposed DL_${\hbox {DA}}_b$ achieves the more competitive performance and outperforms other classification method with augmentation module in most cases.
6. When the data is augmented according to the proportion of the categories in the original data, as shown in Table 3, we can observe that LSTM_${\hbox {DA}}_s$ can achieve the best result. However, our proposed DL_${\hbox {DA}}_s$ achieves the competitive performance and outperforms other classification method with augmentation module in most cases.

**Table 2:** Performance comparisons between the proposed method (DL_${\hbox {DA}}_b$) vs. baselines, using balanced data augmentation.

Methods	All data			Treated			UNTreated		
	Acc	F1	AUC	Acc	F1	AUC	Acc	F1	AUC
LR	0.685	0.628	0.848	0.800	0.550	0.833	0.626	0.601	0.830
PCA_LR	0.362	0.350	0.562	0.420	0.357	0.548	0.333	0.330	0.548
LR_${\hbox {DA}}_b$	0.685	0.619	0.877	0.820	0.563	0.897	0.616	0.587	0.861
SVM	0.745	0.716	0.895	0.840	0.582	0.908	0.697	0.692	0.880
PCA_SVM	0.752	0.723	0.890	0.800	0.651	0.908	0.727	0.720	0.874
SVM_${\hbox {DA}}_b$	0.765	0.744	0.921	0.820	0.563	0.909	0.737	0.733	0.910
MLP	0.745	0.721	0.898	0.820	0.562	0.896	0.707	0.703	0.886
MLP_${\hbox {DA}}_b$	0.812	0.798	0.922	0.860	0.590	0.927	0.788	**0.789**	0.909
LSTM	0.724	0.717	0.892	0.740	0.602	0.824	0.717	0.717	0.905
LSTM_${\hbox {DA}}_b$	0.799	0.777	0.933	0.880	0.763	**0.949**	0.758	0.754	0.923
CNN	0.772	0.757	0.912	0.860	0.589	0.881	0.727	0.728	0.902
CNN_SMOTE	0.798	0.784	0.839	**0.920**	**0.791**	0.877	0.737	0.737	0.805
DL_${\hbox {DA}}_b$	**0.826**	**0.807**	**0.945**	0.900	0.610	0.939	**0.788**	0.786	**0.933**

Best values in each column are bold-faced.

**Table 3:** Performance comparisons between the proposed method method (DL_$\hbox {DA}_s$) against the baselines, using stratified data augmentation (*m*: the augmentation data for each category is *m* as many as the original sample).

Methods	All Data			Treated			UNTreated		
	A	F1	AUC	A	F1	AUC	A	F1	AUC
LR	0.685	0.628	0.848	0.800	0.550	0.833	0.626	0.601	0.830
LR_$${\hbox {DA}}_s$$ ($$m=1)$$	0.671	0.563	0.850	0.840	0.569	0.866	0.586	0.521	0.832
LR_$${\hbox {DA}}_s$$ ($$m=2)$$	0.678	0.589	0.861	0.820	0.556	0.897	0.606	0.557	0.841
SVM	0.745	0.716	0.895	0.840	0.582	0.908	0.697	0.692	0.880
SVM_$${\hbox {DA}}_s$$ ($$m=1)$$	0.691	0.627	0.901	0.820	0.554	0.900	0.626	0.596	0.891
SVM_$${\hbox {DA}}_s$$ ($$m=2)$$	0.725	0.670	0.903	0.820	0.556	0.900	0.677	0.651	0.901
MLP	0.745	0.721	0.898	0.820	0.562	0.896	0.707	0.703	0.886
MLP_$${\hbox {DA}}_s$$ ($$m=1)$$	0.758	0.736	0.904	0.820	0.562	0.893	0.727	0.724	0.891
MLP_$${\hbox {DA}}_s$$ ($$m=2)$$	0.758	0.739	0.910	0.820	0.562	0.905	0.727	0.726	0.896
LSTM	0.724	0.717	0.892	0.740	0.602	0.824	0.717	0.717	0.905
LSTM_$${\hbox {DA}}_s$$ ($$m=1)$$	0.845	0.836	0.934	0.860	0.708	0.880	0.838	0.838	0.937
LSTM_$$\hbox {DA}_s$$ ($$m=2)$$	0.818	0.805	0.952	0.880	0.763	0.963	0.788	0.786	0.940
CNN	0.772	0.757	0.912	0.860	0.589	0.881	0.727	0.728	0.902
DL_$${\hbox {DA}}_s$$ ($$m=1)$$	0.772	0.751	0.922	0.860	0.590	0.861	0.727	0.726	0.910
DL_$${\hbox {DA}}_s$$ ($$m=2)$$	0.785	0.763	0.926	0.840	0.577	0.868	0.758	0.754	0.917

### Parameter analysis

**Impact of the convolution kernel width** *k* In order to study the impact of the convolution kernel width *k*, we use balanced data augmentation by adding $n'=512$ samples to each class, and vary *k* from 3 to 5. The results, reported in Table 4, show that only minor differences are observed using different *k* values. Overall, using $k = 5$, our model DL_$\hbox {DA}_b$ can obtain the best performance.

**Table 4:** Impact of the convolution kernel width *k*, using balanced data augmentation ($n'=512$).

Methods	All Data			Treated			UNTreated		
	Acc	F1	AUC	Acc	F1	AUC	Acc	F1	AUC
CNN ($k=3$)	0.765	0.751	0.904	0.840	0.576	0.865	0.727	0.728	0.898
DL_${\hbox {DA}}_b$ (*k*=3)	0.812	0.791	0.939	0.900	0.610	0.930	0.768	0.766	0.928
CNN ($k=4$)	0.765	0.751	0.910	0.840	0.576	0.873	0.727	0.728	0.901
DL_${\hbox {DA}}_b$ (*k*=4)	0.826	0.806	0.943	0.900	0.610	0.934	0.788	0.786	0.931
CNN ($k=5$)	0.772	0.757	0.912	0.860	0.589	0.881	0.727	0.728	0.902
DL_${\hbox {DA}}_b$ ($k=5$)	**0.826**	**0.807**	**0.945**	**0.900**	**0.610**	**0.939**	**0.788**	**0.786**	**0.933**

Best value in each column is bold-faced.

**Impact of the sample size **$n'$

- **Balanced data augmentation** For balanced data augmentation, we set the augmentation sample size $n'$ to 128, 256, 512, 1024, respectively, and report the results in Table 5. The results show that as the augmentation sample size $n'$ increases, the accuracy continues to improve, but when the number of $n'$ increases to 1024, the various evaluation indicators will decrease, which may be due to the imbalance between the original data and the augmentation data.

**Table 5:** Impact of the data augmentation sample size $n'$, using balanced data augmentation and kernel width $k=5$.

Methods	All Data			Treated			UNTreated		
	Acc	F1	AUC	Acc	F1	AUC	Acc	F1	AUC
DL_${\hbox {DA}}_b$ ($n'=128$)	0.812	0.796	0.929	0.880	0.596	0.868	0.777	0.776	0.920
DL_${\hbox {DA}}_b$ ($n'=256$)	0.799	0.778	0.935	0.900	0.610	0.908	0.748	0.747	0.923
DL_${\hbox {DA}}_b$ ($n'=512$)	**0.826**	**0.807**	0.945	**0.900**	**0.610**	**0.939**	**0.788**	**0.786**	0.933
DL_${\hbox {DA}}_b$ ($n'=1024$)	0.812	0.793	**0.946**	0.880	0.610	0.923	0.778	0.776	**0.936**

Best value in each column is bold-faced.
- **Stratified data augmentation** For stratified data augmentation, we set the *m* to 1, 2, 4, 6 and 8. For example, when $m=2$, the data augmentation module will generate $2*36$, $2*63$, $2*50$ augmentation samples for the BCC, NORMAL and SCC dataset, respectively, and their training samples will increase to $2*36+36$, $2*63+63$ and $2*50+50$, respectively. We report the results in Table 6. The results show as the number of the *m* increases, the classification accuracy continues to improve, but when the number of *m* increases to 8, the various evaluation indicators will sightly decrease.

**Table 6:** Impact of the data augmentation sample size $n'$, using stratified data augmentation and kernel width $k=5$ (*m* represents that the augmentation data for each category is *m* as many as the original sample).

Methods	All Data			Treated			UNTreated		
	A	F1	AUC	A	F1	AUC	A	F1	AUC
DL_${\hbox {DA}}_s$ ($m=1$)	0.772	0.751	0.922	0.860	0.590	0.861	0.727	0.726	0.910
DL_${\hbox {DA}}_s$ ($m=2$)	0.785	0.763	0.926	0.840	0.577	0.868	0.758	0.754	0.917
DL_${\hbox {DA}}_s$ ($m=4$)	0.805	0.787	0.936	0.880	0.604	0.886	0.768	0.767	0.927
**DL**_${\hbox {DA}}_s$ ($m=6$)	**0.832**	**0.816**	**0.944**	0.900	0.610	**0.912**	**0.798**	**0.797**	**0.935**
DL_$\hbox {DA}_s$ ($m=8$)	0.825	0.813	0.928	**0.920**	**0.625**	0.905	0.778	0.778	0.911

Best value in each column is bold-faced.

**Impact of the number of filters ***r* In order to to study the impact of the number of filters *r*, we set the number of filters *r* to 60, 120, 180, respectively, and report the result in Table 7. The results show that as the number of filters *r* increases, the classification accuracy remains stable and does not change significantly.

**Table 7:** Impact of the number of filters *r*, using kernel width $k=5$ and no data augmentation.

Methods	All Data			Treated			UNTreated		
	A	F1	AUC	A	F1	AUC	A	F1	AUC
CNN ($r=16$)	0.765	0.753	0.813	0.840	0.576	0.740	0.727	0.728	0.902
CNN ($r=32$)	0.765	0.747	0.812	0.860	0.589	0.881	0.717	0.717	0.902
CNN ($r=120$)	0.772	0.757	0.912	0.860	0.589	0.881	0.727	0.728	0.902
CNN ($r=180$)	0.772	0.757	0.912	0.860	0.589	0.881	0.727	0.728	0.902
CNN ($r=256$)	0.785	0.769	0.825	0.860	0.589	0.881	0.737	0.737	0.804
CNN ($r=512$)	0.778	0.762	0.821	0.860	0.589	0.881	0.737	0.737	0.804

### Case study

#### Data distribution visualization

In order to verify the effectiveness of the augmentation module of our model, we visualize the original data and new samples generated by the generative adversarial network.

**Figure 5:**
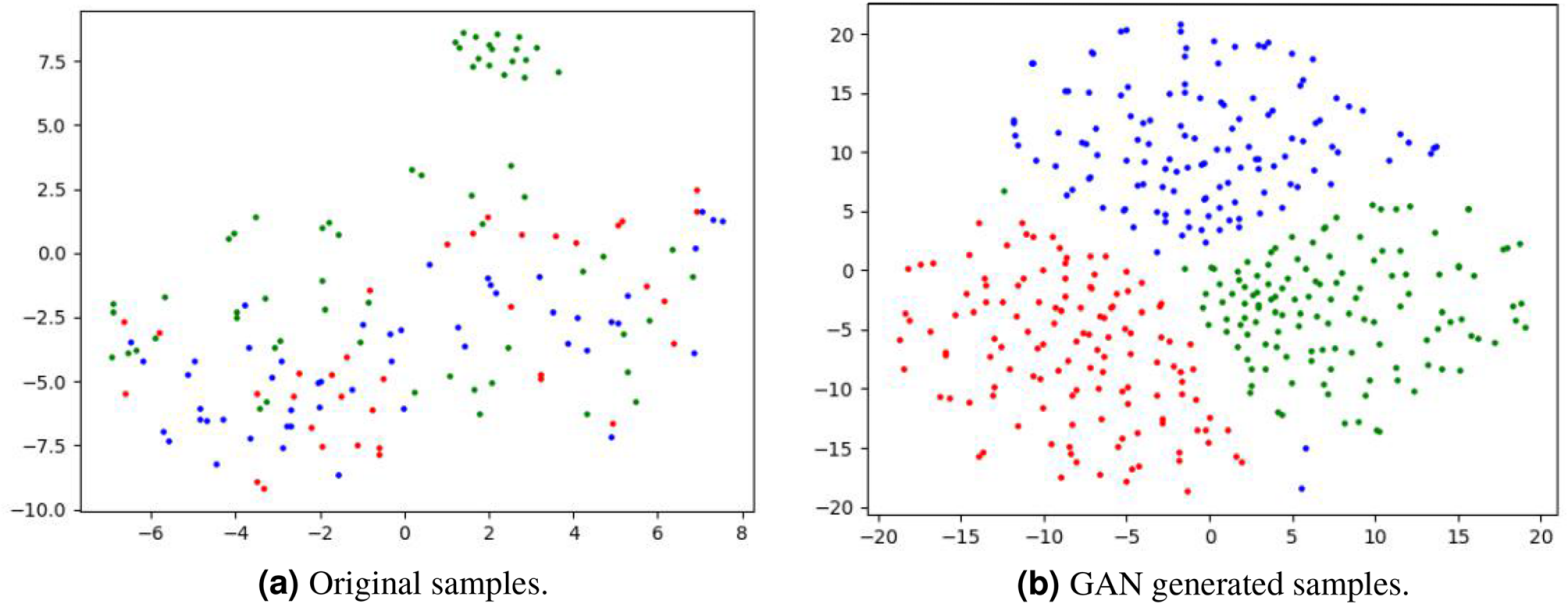
Visualization of the distributions of the original samples (**a**) vs. GAN generated samples (**b**), in the RS spectra feature space using *t*-SNE^30^. Each dot denotes a sample, color-coded by the label where red, blue, green denote BCC, SCC, Normal, respectively (using balanced data augmentation with $n'=128$).

From Fig. 5a, we can observe that the distribution of the original data is more scattered and difficult to distinguish, due to the small amount of data. Otherwise, from Fig. 5b, we can observe that compared with original samples, different class of samples generated by GAN are easy to distinguish (we use balanced data augmentation with $n'=128$). The results show that generated samples not only expand the coverage of training dataset, but also preserve the key feature distributions of the original data.

#### The confusion matrix

**Figure 6:**
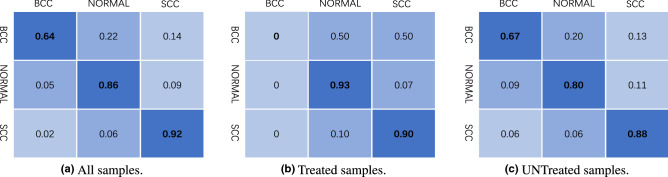
The confusion matrix of the proposed DL_DA.

In order to verify the effectiveness of DL_DA in differentiating diffident class samples, Fig. 6 reports the confusion matrix of DL_DA on all data (we use balanced data augmentation, using $n'=128$ and kernel size $k=5$). The results show that DL_DA remains a high accuracy in separating different types of tissue samples.

### Discussion

There are three possible reasons explaining why data enhancment module proposed in the paper is more effective than other models: (1) increased sample density; (2) increased sample diversity; and (3) increased resemblance between synthetic vs. genuine samples. All of which have helped learn better decision boundaries for separation.

For increased sample density, data augmentation can generate more samples, similar to the training data, and help increase the training set density. With a higher density, the classifiers can often learn more precise boundaries, compared to sparse data. This explains why data augmentation often outperforms classifiers learned from original sample set.

For increased sample diversity, deep learning data augmentation is essentially different from bagging (which duplicates training samples) or SMOTE (which uses linear space conversion to create new features). The non-linear transformation used in deep learning allows more diverse samples to be generated.

For increased resemblance between synthetic vs. genuine samples, the adversarial learning process (between generator and discriminator) ensures that synthetic samples are very similar, but not identical, to the original training data. This increases the resemblance of synthetic sample set to the original training set.

## Conclusions

In this paper, we proposed to use deep learning for Raman Spectroscopy based cancer tissue classification, by using data augmentation method to increase the number of training samples in order to enhance downstream classifiers for better feature representation learning from RS signals and better classification. To achieve the goal, we proposed a novel generative adversarial network based skin cancer tissue classification framework with two major components: (1) Data augmentation module, and (2) Data classification module. The former employs a Generative Adversarial Network for data augmentation to obtain a sufficient number of data, and the latter uses five different approaches for classification. Our study validates different ways of data augmentations, including balanced data augmentation to add same number of samples to each class and stratified data augmentation to preserve class distributions. Experiment results show that GAN can be successfully utilized for augmenting data and generating synthetic samples resemble to the original samples. Moreover, the proposed DL_DA (using GAN to generate augmentation samples and CNN for final classification) obtains the best performance to separate BCC, and SCC cancer, from normal samples.

Due to resource constraints, our study is only based on a rather small RS sample set. We are working towards obtaining additional RS samples from different sources for validation. Using other data augmentation approaches, such as contrastive learning, for feature learning from RS data is also a future direction of our study.
